# Transfer mechanism of cell-free synthesized membrane proteins into mammalian cells

**DOI:** 10.3389/fbioe.2022.906295

**Published:** 2022-07-22

**Authors:** Simon Umbach, Roman Levin, Sebastian Neumann, Torsten Steinmetzer, Volker Dötsch, Frank Bernhard

**Affiliations:** ^1^ Institute of Biophysical Chemistry and Center for Biomolecular Magnetic Resonance, Goethe University, Frankfurt am Main, Germany; ^2^ Institute for Pharmaceutical Chemistry, Philipps University, Marburg, Germany

**Keywords:** G-protein coupled receptors, cell-free expression, nanodiscs, Salipro nanoparticles, transfection, protein transfer, GPCR function, HEK 293 cell

## Abstract

Nanodiscs are emerging to serve as transfer vectors for the insertion of recombinant membrane proteins into membranes of living cells. In combination with cell-free expression technologies, this novel process opens new perspectives to analyze the effects of even problematic targets such as toxic, hard-to-express, or artificially modified membrane proteins in complex cellular environments of different cell lines. Furthermore, transferred cells must not be genetically engineered and primary cell lines or cancer cells could be implemented as well. We have systematically analyzed the basic parameters of the nanotransfer approach and compared the transfer efficiencies from nanodiscs with that from Salipro particles. The transfer of five membrane proteins was analyzed: the prokaryotic proton pump proteorhodopsin, the human class A family G-protein coupled receptors for endothelin type B, prostacyclin, free fatty acids type 2, and the orphan GPRC5B receptor as a class C family member. The membrane proteins were cell-free synthesized with a detergent-free strategy by their cotranslational insertion into preformed nanoparticles containing defined lipid environments. The purified membrane protein/nanoparticles were then incubated with mammalian cells. We demonstrate that nanodiscs disassemble and only lipids and membrane proteins, not the scaffold protein, are transferred into cell membranes. The process is detectable within minutes, independent of the nanoparticle lipid composition, and the transfer efficiency directly correlates with the membrane protein concentration in the transfer mixture and with the incubation time. Transferred membrane proteins insert in both orientations, N-terminus in and N-terminus out, in the cell membrane, and the ratio can be modulated by engineering. The viability of cells is not notably affected by the transfer procedure, and transferred membrane proteins stay detectable in the cell membrane for up to 3 days. Transferred G-protein coupled receptors retained their functionality in the cell environment as shown by ligand binding, induction of internalization, and specific protein interactions. In comparison to transfection, the cellular membrane protein concentration is better controllable and more uniformly distributed within the analyzed cell population. A further notable difference to transfection is the accumulation of transferred membrane proteins in clusters, presumably determined by microdomain structures in the cell membranes.

## 1 Introduction

The *in vivo* analysis of membrane proteins (MPs) in cellular systems frequently suffers from low endogenous concentrations, unknown regulation mechanisms, or heterogeneous expression levels in a cell population. Recombinant MP production by genetic tools such as DNA or RNA transfection can address these issues, but still, a variety of problems such as MP toxicity by blocking translocon systems, misfolding, wrong trafficking, or failure to insert into cellular membranes may occur (Kim and Eberwine, 2010). Furthermore, artificial modifications of MPs by directed engineering, by attachment of probes for monitoring, or by modulation of MP complexes can be difficult or even hardly possible. The direct transfer of purified MPs into live cells could eliminate many problems and would be a straightforward approach to analyzing the *in vivo* effects of designed MPs by potentially having better control on protein quality, modifications, and copy number per cell.

The cotranslational insertion of MPs into nanodisc (ND) membranes by cell-free (CF) expression is an efficient method to produce a variety of functional MPs including G-protein coupled receptors (GPCRs), transporters, channels, or enzymes (Henrich et al., 2016; Henrich et al., 2017b; He et al., 2017; Rues et al., 2018; Keller et al., 2019; Kuroiwa et al., 2022). The NDs are first pre-assembled with the lipid or lipid mixture that is best suited for the stability and folding of the selected MP. CF expression reactions of the MP are then performed in presence of suitable concentrations of the supplied NDs (Henrich et al., 2016; Peetz et al., 2017). The technique gives fast access to even difficult proteins and MP/ND samples can be generated within 24 h. As no live cells are involved, any potentially toxic effects during MP production are eliminated. In addition, the MPs do not have contacts with destabilizing detergents during the whole synthesis and purification process. The open nature of CF systems further enables a high potential to modify the synthesized MPs, e.g., by a variety of engineering approaches, by attachment of probes, or by complex formation with artificial ligands.

A continuously growing toolkit for the transfer of soluble proteins into live cells by electroporation, direct injection, inclusion into liposomes, or by using cell-penetrating peptides is available (Chau et al., 2020). In contrast, the transfer of MPs is much less explored despite their high importance for pharmaceutical research (Overington et al., 2006). A problematic issue is the requirement of a suitable membrane mimetic to keep the MP soluble and stable while also being compatible with the transfer approach (Majeed et al., 2021). A significant advantage for MP transfer was the implementation of NDs as vectors (Denisov and Sligar, 2017). If NDs are mixed with other lipid bilayers such as micelles, supported bilayers, or even lipid cubic phases, lipids and inserted MPs can be transferred (Lai et al., 2015; Henrich et al., 2017a; Nikolaev et al., 2017; Dang et al., 2019). Furthermore, NDs have been used to transfer a variety of other biomolecules such as RNA, optical probes, or drugs into living cells (Murakami, 2012; Carney et al., 2015; He et al., 2020).

The successful nanotransfer of CF synthesized MPs, such as the human ß2-adrenergic receptor and the Her2 receptor tyrosine kinase, from NDs into live cells was already demonstrated (Patriarchi et al., 2018; He et al., 2021). This new process has a high potential to efficiently close a gap between the *in vitro* and *in vivo* analysis of MPs. Aliquots of CF-generated MP/ND samples can simultaneously be analyzed *in vitro* as well as in cell-based assays. During the complete procedure, the MPs stay in lipid environments and *in vitro* functions in defined lipid bilayers can be compared with those in the much more complex environment of living cells.

Mechanism and parameters important for the modulation of an efficient MP transfer from NDs into cells are still mostly unknown. Furthermore, details about the membrane topology and stability of transferred MPs are lacking. We have studied kinetic details of the MP transfer mechanism and analyzed the effect of key compounds such as the type of nanoparticle, lipid composition, or cell line on the transfer efficiency. As examples for human class A GPCRs, we have selected the endothelin B receptor (ETB), the prostacyclin receptor (IP), and the free fatty acid receptor 2 (FFAR2). In addition, the human orphan receptor GPRC5B as a member of the class C family of GPCRs and the seven-transmembrane domain-containing proton pump proteorhodopsin (PR) were selected as model MPs. We demonstrate that the functional nanotransfer of CF-synthesized MPs from NDs and also from Salipro particles into cellular membranes is a rather universal mechanism. We show the functionality of transferred GPCRs in mammalian cells and compare their localization with that of GPCR controls synthesized by transfection. The orientation of transferred MPs in the cell membrane was analyzed and modulated by engineering. As a particular feature of the nanotransfer approach, we first demonstrate that in contrast to MP production in transfected cells, a significant fraction of the transferred MPs is organized in membrane-located clusters.

## 2 Methods

*DNA constructs and ligands*: sequences encoding for human GPCRs (Supplementary Table S1) were codon-optimized for *E. coli* and flanked by an N-terminal Htag for improved CF expression yields and a C-terminal Strep-tag for purification and detection (Haberstock et al., 2012). ETB constructs were based on the thermostabilized Y5 mutant and synthesized as published in some studies (Okuta et al., 2016; Rues et al., 2018). To monitor the nanotransfer, GPCR-mNeonGreen (mNG) fusion constructs were created by subcloning the mNG sequence downstream of the GPCR sequences via KpnI and XhoI restriction sites. For immunostaining experiments, a Myc-tag was inserted either between the Htag and the GPCR or between the GPCR and the Strep-tag using a standard quick-change site-directed mutagenesis approach. Flag-tagged MSP1E3FN was created by inserting the Flag-sequence between the N-terminal His6-TEV sequence and the MSP gene via quick change. This enables the removal of the His-tag after protein expression and purification, resulting in an N-terminally Flag-tagged MSP.

The circular ligand cET-1 for the endothelin type B receptor was obtained as described in Proverbio et al. (2013). The synthesis of biotinylated 4-Ala-ET-1 and DY647-4-Ala-Arg9-ET-1 is described in detail in the supplementary materials section.

*Nanoparticles*: NDs were performed with purified membrane scaffold protein (MSP) as described in previous studies (Rues et al., 2018; Köck et al., 2021). If not indicated otherwise, MSP1E3D1 was used for ND preparation, resulting in NDs with a diameter of 10–12 nm, which was a suitable compromise between lipid bilayer size and stability, since NDs with larger MSPs tend to be less stable. MSP1E3D1:lipid ratios were 1:80 (DEPG), 1:80 (DOPG), 1:90 (POPG) and 1:115 (DMPC). Rho-PE containing NDs was prepared by adding 2% (lissamine rhodamine B)-PE to DEPG before ND formation.

For SapA nanoparticle (SapNP) preparation, DOPG liposomes were prepared by first solubilization of DOPG in chloroform followed by vacuum evaporation. The dried lipid bilayers were then suspended in 50 mM Tris-HCl, pH 8.0, 50 mM NaCl, and adjusted to a 30 mg/ml suspension. SapA was purified as described elsewhere (Frauenfeld et al., 2016) and concentrated to 20–30 mg/ml (1.7–2.6 mM). Subsequently, SapA solution and lipid suspension were mixed to yield a stoichiometric ratio of 1:24 SapA:DOPG. To induce particle formation, pH was shifted to 4.8–5.0 using 5% (v/v) acetic acid. Upon acidification, the turbid suspension was clarified and the solution was then rapidly diluted 1:10 with 50 mM Tris-HCl, pH 8.0, 50 mM NaCl to neutralize pH. The solution was then centrifuged at 30,000 x g for 20 min to remove insoluble material and the final concentration of formed particles was estimated via SapA concentration by UV-spectroscopy with ε = 10.345 M^−1^ cm^−1^ for non-TEV treated SapA. Total protein concentration should be 20–30 mg/ml (1.7–2.6 mM) to yield 400 µM total SapA in the CF mix which corresponds to 100–200 µM total SapNPs, considering that one particle is assembled with 2-4 SapA conformers.

*CF expression*: Two-compartment CF expression was based on *E. coli* A19 S30 lysate and performed as described in previous studies (Schwarz et al., 2007; Köck et al., 2021). Briefly, a semipermeable membrane (12–14 kDa cut-off) is used to separate a reaction mixture (RM) from a feeding mixture (FM). The RM contains all the necessary components for transcription and translation. The FM provides additional amino acids and energy sources and helps to dilute inhibitory by-products from the RM. Typically, reaction containers with 55 µl RM and 800 µl FM were used. Larger reaction volumes (1–3 ml RM) were incubated in dialysis cassettes. The final Mg^2+^ concentration was adjusted to 18 mM. Depending on the receptor, either DTT (final concentration 2 mM) or a 3:1 mixture of reduced and oxidized glutathione (GSH/GSSG; final concentration 3 and 1 mM) was used as a redox system. If not indicated otherwise, 60 µM preformed NDs were used in the RM containing lipids found to be suitable for the GPCR integration (Rues et al., 2018): DEPG (ETB and IP derivatives) and POPG (FFAR2 and GPRC5B derivatives). Different lipids were used for PR as indicated in the results section. Expression was performed overnight at 30°C under slight shaking. Afterward, precipitates were removed via centrifugation for 10 min at 18,000 x g and the synthesized MP/nanoparticle complexes were purified via the Strep-tag of the MP from the supernatant.

*Protein purification and analysis*: receptors were purified from the RM by Strep-Tactin affinity chromatography using a gravity flow column equilibrated in purification buffer (100 mM Tris-HCl, pH 8.0, 100 mM NaCl). Elution was done with *d*-desthiobiotin (25 mM, in purification buffer), which was subsequently removed through washing in Amicon centrifugal filters (50 kDa MWCO).

Size exclusion chromatography (SEC) was used for characterization and purification of the synthesized MPs. SEC was performed using a Superose 6 increase 3.2/300 column. For preparative SEC, samples were fractionated and fractions of interest were pooled and concentrated. For ligand binding and activity studies of ETB-mNG, purification was done via ligand affinity chromatography (LAC) using immobilized biotinylated 4Ala-ET-1. Therefore, biotinylated 4Ala-ET-1 in binding buffer was incubated with magnetic Strep-Tactin beads (MagStrep “type3” XT, IBA) overnight. ETB-mNG was bound for 1 h at 4°C while gently mixing by inversion. The beads were washed with binding buffer and the complex was eluted using a 10-fold excess of 4Ala-ET-1. Receptor-bound 4Ala-ET-1 was then removed by extensive washing in centrifugal filters. After purification, samples were concentrated in centrifugal filters and protein concentration was determined either via mNG fluorescence or via nanodrop measurements for receptors without fluorophore. Concentrations were calculated assuming a single receptor per ND.

For western blotting, samples were loaded onto a 4–15% continuous Mini-PROTEAN TGX gel (BioRad) and separated for 30 min at 200 V. Blotting was done using the Trans-Blot Turbo System (BioRad). Membranes were blocked with 4% skim milk powder for 1 h at room temperature. Primary antibodies (anti-Myc (1:2000, 4A6, Millipore); anti-HA (1:1,000, A190-138A, Bethyl); anti-His (1:2,000, H1029)) were applied for 1 hour. After washing, secondary antibodies (anti-mouse-HRP (1:5,000, A9917, Sigma)) were applied for another hour at RT. For the detection of Strep-tagged proteins, an anti-Strep HRP conjugate was used (1:5,000; #1610381, BioRad). All antibody incubation and washing steps were done in PBS-T (0.05% Tween-20). Chemiluminescence was analyzed using a Lumi-Imager F1 (Roche).

*Cell culture*: CHO-K1, HEK293T, and H1299 cells were grown in Ham’s F-12 medium, DMEM and RPMI, respectively. All media were supplemented with 10% fetal calf serum and 1% penicillin/streptomycin. All cell lines were grown at 37°C and 5% CO_2_ and tested regularly negative for *mycoplasma* contamination. Transfections were performed using the Lipofectamine 2000 kit (ThermoFisher). For fluorescence microscopy, cells were seeded at a density of 2 × 10^5^ cells/well onto glass coverslips in a 12-well plate. To prevent detachment of cells during washing steps, the medium for HEK293T cells was supplemented with 0.2 μg/ml poly-l-lysine.

*Nanotransfer and confocal fluorescence microscopy*: for nanotransfer experiments, cells seeded on coverslips in 12-well plates were washed once with 1 mL Dulbecco’s phosphate-buffered saline (DPBS) and then incubated with 500 µL standard media supplemented with a defined concentration of nanoparticles. Cells were then kept at 37°C and 5% CO_2_. After incubation, cells were washed five times with 1 mL DPBS and fixed for 10 min in RotiHistofix. After three additional washing steps to remove the residual fixation agent, coverslips were placed on microscope slides with a DAPI-containing mounting medium. If not stated otherwise, 0.5 µM nanoparticles and 4 h of incubation were used as standard conditions for all nanotransfer experiments.

Immunostainings were carried out by permeabilizing cells after fixation with 0.2% Triton X-100 in DPBS for 20 min. After three washing steps, cells were blocked for 1 h with DPBS containing 1% BSA. Primary antibodies (anti-Myc (1:100, 4A6, Millipore); anti-Flag (1:100, F3165, Sigma)) were added for 1 h. Cells were washed again and the secondary antibody (anti-mouse Alexa647 conjugate (1:500, A31571, life technologies)) was added. After washing, cells were mounted. All antibody incubation and washing steps were carried out in DPBS with 1% BSA at RT.

To evaluate ligand binding of ETB derivatives, HEK293T cells were washed five times with DPBS after transfer of LAC-purified ETB-mNG, and fresh media containing 100 nM DY647-ET-1 was added. Cells were incubated for 1 h at 4°C to prevent internalization. Afterward, they were washed with DPBS and mounted. For the internalization assay, cells were incubated with cET-1 for 1 h at 4°C. After ligand incubation, cells were transferred back to 37°C for 1 h before mounting.

Images were acquired using an inverted Zeiss Observer Z1 with a Yokogawa CSU-X1A 5000 spinning disc unit and an EMCCD camera. Imaging was done through a ×63 oil objective. Four lasers of 405, 488, 561, and 638 nm with corresponding 450/50, 485/30, 562/45, 690/50 nm bandpass filters for blue-, green-, orange-, and red-emitting fluorophores, respectively. Images were quantified using ImageJ. Membrane fluorescence intensity was calculated by tracing the plasma membrane of a number of randomly picked cells.

*Co-IP of transfected and delivered GPCRs*: for Co-IP experiments, HEK293T cells were seeded into 6-well plates at a density of 1.5 × 10^6^ cells/well. After 24 h, cells were transfected with 2 µg per well plasmid DNA encoding for either GPRC5B-Myc or HA-IP. 6 h after transfection, the culture medium was removed and a fresh medium containing 0.5 µM nanoparticles with or without GPCR was added. After 16 h, cells were washed five times with ice-cold DPBS and lysed in lysis buffer (25 mM Tris-HCl, pH 7.4, 150 mM NaCl, 1% NP-40, 1 mM EDTA, 5% glycerol) supplemented with complete protease inhibitor for 30 min on ice. The lysate was centrifuged at 22,000 × g for 15 min to remove cellular debris. The cleared lysate was then incubated with magnetic Strep-Tactin beads (MagStrep “type3” XT, IBA) for 2 h at 4°C under constant rotation. Afterward, beads were washed three times with ice-cold lysis buffer. SDS loading buffer was added and the sample was incubated at 95°C for 10 min before SDS-PAGE and western blot.

## 3 Results

### 3.1 CF production and preparation of MP/ND samples

**FIGURE 1:**
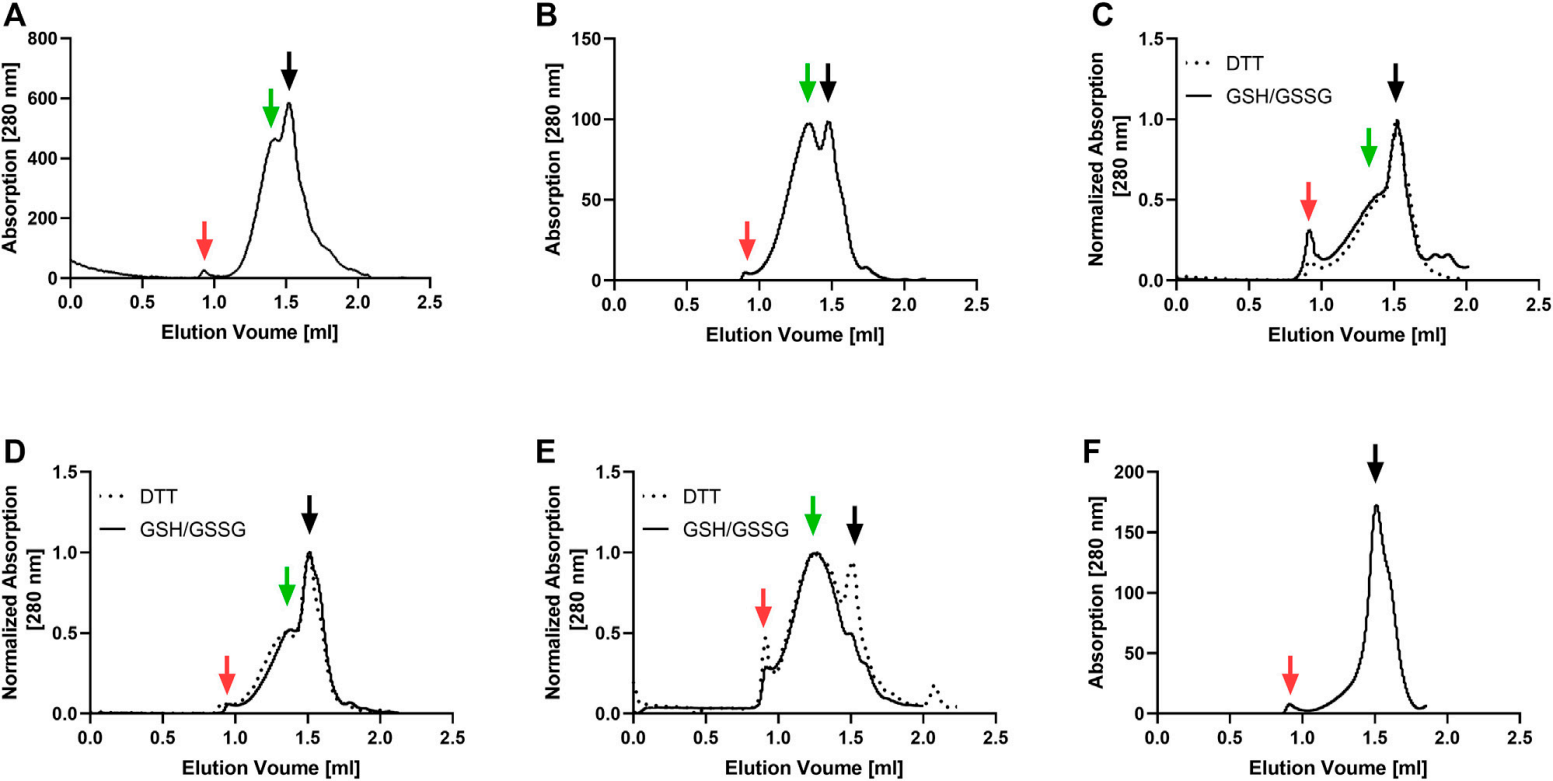
SEC profiling of MP/ND samples used for nanotransfer into mammalian cells. The receptors were cotranslationally inserted into preformed NDs, Strep-purified and analyzed with a Superose 6 increase 3.2/300 column. Solid and dotted lines correspond to elution profiles of GPCR/ND samples CF synthesized in presence of either a GSH/GSSG or a DTT redox system. **(A)** ETB **(B)** ETB-tc; **(C)** FFAR2; **(D)** GPRC5B; **(E)** IP; **(F)** PR. Void peak (red), fraction 2 (black) and fraction 1 (green) are indicated with arrows.

The GPCRs and PR were synthesized in an *E. coli*-based CF system and cotranslationally inserted into preformed NDs assembled with lipids found to be most suitable for the stability of the selected MPs. The production efficiency of the MP/ND complexes was between 0.7 and 1.1 mg/ml of RM for all analyzed targets. MP/ND complexes were purified via the C-terminal Strep-tag of the MPs. SEC profiling of the purified MP/ND complexes was used to evaluate the sample homogeneity (Figure 1). With all GPCR samples, two major SEC fractions were detectable. While fraction 2 is assumed to contain homogenous MP/ND particles, fraction 1 may include MP/ND aggregates. With PR, the most homogeneous profile, showing only fraction 2 was observed. GPCRs contain at least one disulfide bridge essential for their functional folding and thus the redox conditions during CF expression were optimized. ETB and ETB-tc were synthesized in presence of the redox system GSH/GSSG according to previously established protocols (Dong et al., 2018). GPRC5B/ND, FFAR2/ND, and IP/ND particles have not been CF synthesized before and their quality was therefore analyzed after CF expression in presence of either DTT or GSH/GSSG as a redox system (Figure 1). A significant improvement of IP/ND homogeneity toward fraction 2 was monitored after synthesis in presence of DTT. With GPRC5B/NDs and FFAR2/NDs, no differences in the SEC profiles after production in the two redox systems could be detected.

### 3.2 Basic parameters of ND-mediated MP transfer into mammalian cells

**FIGURE 2:**
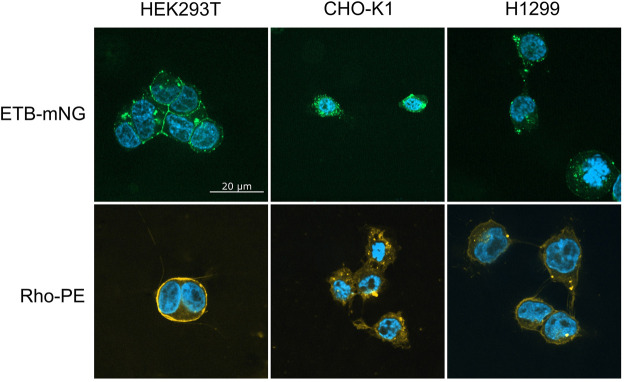
Transfer of ETB-mNG and Rho-PE from NDs into different mammalian cell lines. 0.5 µM of NDs (DEPG) containing either ETB-mNG or 2% Rho-PE were incubated for 4 h with HEK293T, CHO-K1, or H1299 cells. After washing and fixation, cells were analyzed using a spinning disk fluorescence microscope. Representative images are shown.

In order to better understand the fundamental principles and mechanisms of the nanotransfer approach, a number of potentially relevant parameters including different cell lines and culture conditions as well as modified MPs and various ND lipid compositions were analyzed. First, 0.5 µM NDs (DEPG) containing either ETB-mNG or 2% Rho-PE lipids were incubated with HEK293T, CHO-K1, or H1299 cells for 4 h at 37°C. After washing and fixation, the cells were analyzed by fluorescence microscopy (Figure 2). In HEK293T cells, the transferred ETB-mNG and Rho-PE were the most evenly distributed in the plasma membrane, whereas in H1299 and in particular in CHO-K1 cells fluorescent spots appear to be more localized within the cytoplasm. Fluorescence quantification of representative ETB-mNG transferred cells further confirmed the primary localization of ETB-mNG in the HEK293T plasma membrane (Supplementary Figure S1). Notably, some cluster formation of the transferred ETB-mNG in the HEK293T membrane can be observed, while the distribution of the Rho-PE lipid appears to be more evenly. Based on the more preferential localization of the transferred compounds in the cell membrane, HEK293T cells were selected for further experiments. The incubation with 0.5 μM MP/ND particles for 4 h at 37°C were taken as standard conditions. The transfer of ETB-mNG into HEK293T cells was unaffected by the presence of either antibiotics or FCS in the medium (data not shown).

**FIGURE 3:**
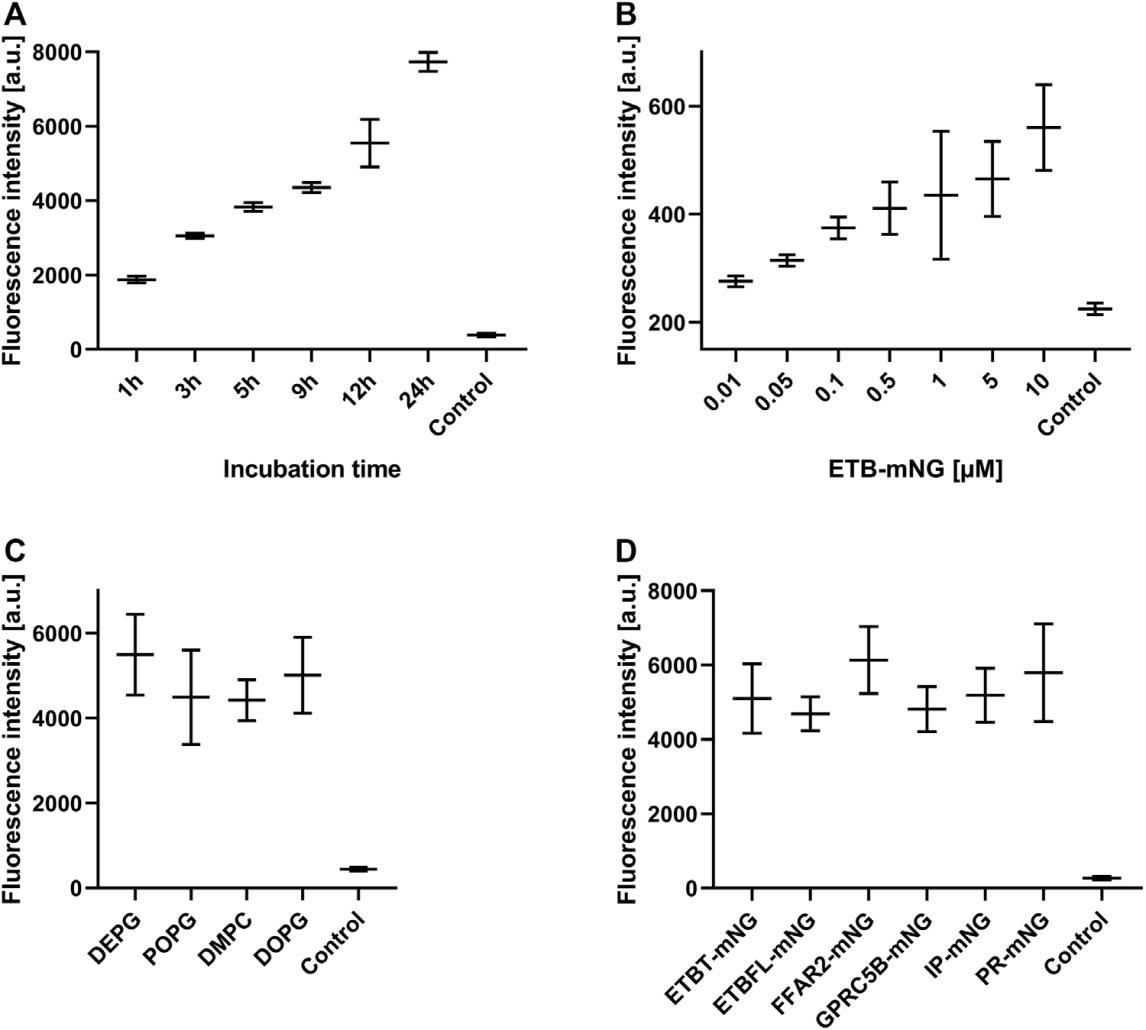
Evaluation of MP transfer efficiency into HEK293T cells. HEK293T cells were incubated with Strep-purified MP/ND particles at standard conditions (0.5 μM MP/ND particles, 4 h transfer) with the indicated modifications. After transfer, cells were fixed, and membrane fluorescence was quantified. **(A)** Effect of incubation times on ETB-mNG transfer. **(B)** Effect of ETB-mNG/ND particle concentration. **(C)** Transfer of PR-mNG from NDs containing either DEPG, POPG, DMPC, or DOPG lipids. **(D)** Transfer of GPCR-mNG and PR-mNG fusions from NDs containing customized membrane compositions (ETB-mNG/ND (DEPG), ETB-tc-mNG/ND (DEPG), FFAR2-mNG/NDs (POPG), GPRC5B-mNG/NDs (POPG), IP-mNG/NDs (DEPG) and PR-mNG/NDs (DEPG)). Mean ± SEM is shown. N = 20 cells. Controls are fluorescence of HEK293T cells after incubation with NDs without MPs.

Next, the effect of MP/ND concentration and incubation time on the transfer efficiency was analyzed (Figure 3, Supplementary Figure S2). Transfer by incubation of 0.5 µM ETB-mNG/ND with HEK293T cells for a period from 1 to 24 h was analyzed by quantification of membrane fluorescence (Figure 3A). The detection of ETB-mNG in the cell membrane increased linearly with the incubation time for the whole period. The concentration of ETB-mNG/ND particles in the incubation mixture was next screened from 10 nM to 10 µM by incubation for 4 h at 37°C (Figure 3B). Already with the lowest concentration of 10 nM, a transfer of the receptor into the cell membranes was detectable (Figure 3B). In a complementary experiment, the transfer of lipids from NDs (DEPG +2% Rho-PE) into HEK293T cells was analyzed within a concentration range of 50 nM to 5 µM NDs and incubation times from 1 to 24 h (Supplementary Figure S3). Similar to the MP transfer, the transfer of lipids was fast and detectable already with 50 nM NDs. The lipid transfer also increased linearly with ND concentration and incubation time.

The membrane composition of NDs can be crucial for the folding and stability of inserted GPCRs (Rues et al., 2018) and might also affect the transfer efficiency. PR-mNG/ND particles were prepared with the lipids—DEPG, POPG, DMPC, or DOPG—and subsequently transferred at standard conditions. Fluorescence analysis revealed no major differences in the transfer efficiency of PR-mNG from the different lipid environments into HEK293T cells (Figure 3C). This would allow to transfer individual MPs inserted into their most suitable lipid environment. A variety of GPCRs inserted into ND membranes of specific lipid compositions were then analyzed for their transfer efficiency into HEK293T cell membranes. Samples of ETB-mNG/ND (DEPG), ETB-tc-mNG/ND (DEPG), FFAR2-mNG/NDs (POPG), GPRC5B-mNG/NDs (POPG), IP-mNG/NDs (DEPG) and PR-mNG/NDs (DEPG) were incubated with HEK293T cells at standard conditions and the membrane fluorescence was quantified (Figure 3D). The overall transfer efficiency of all analyzed MPs was found to be similar, indicating that a wider range of MPs inserted in customized lipid environments can be used as a target for the nanotransfer approach.

### 3.3 NDs disintegrate upon MP transfer

**FIGURE 4:**
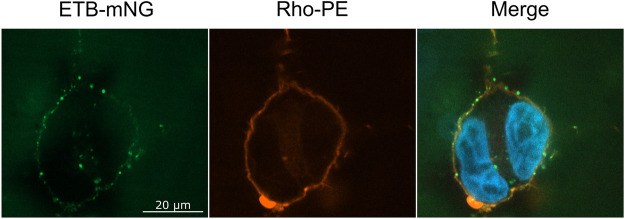
Localization of ETB-mNG and Rho-PE lipids after co-transfer into HEK293T cells. Purified ETB-mNG/NDs (DEPG +2% Rho-PE) were incubated with HEK293T cells at standard conditions. After fixation, the localization of transferred ETB-mNG and Rho-PE in the cells was analyzed via fluorescence microscopy. Representative images are shown.

**FIGURE 5:**
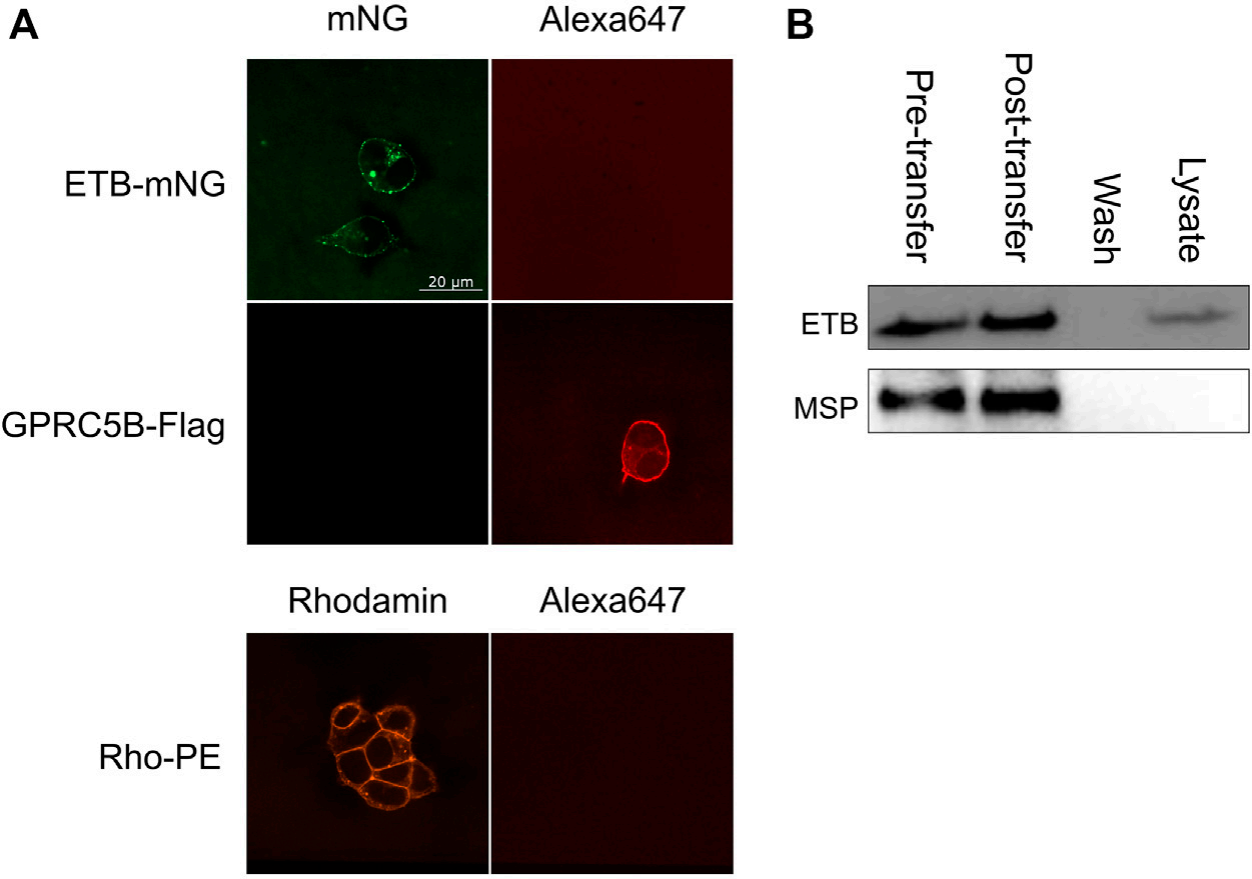
Analysis of MSP transfer into HEK293T cells. **(A)** ETB-mNG/NDs and NDs (DEPG +2% Rho-PE) performed with Flag-tagged MSP1E3FN were incubated with HEK293T cells at standard conditions. Cells were then washed, fixed, permeabilized, and incubated with anti-Flag primary antibodies and Alexa647 coupled secondary antibodies. Transfected HEK293T cells synthesizing GPRC5B-Flag were used as control. **(B)** ETB/NDs were incubated with HEK293T cells at standard conditions and transfer of ETB and MSP was monitored by western blotting with anti-Strep (ETB) and anti-His (MSP) antibodies, respectively. Pre-transfer: Samples of the transfer mixture before incubation; post-transfer: Samples of the transfer mixture after incubation; wash: DPBS after the last washing step; lysate: Cell lysate after incubation and washing.

The NDs may disintegrate upon transfer or at least some fraction may stay intact and attaches to the cellular membrane or may even become incorporated into the cell. To address this question, ETB-mNG was CF inserted into NDs (DEPG) containing 2% Rho-PE lipids. The resulting purified ETB-mNG/Rho-PE/NDs were then incubated with HEK293T cells under standard conditions and analyzed by fluorescence microscopy (Figure 4). A substantial fraction of transferred ETB-mNG again localized in clusters, while the Rho-PE lipids distributed homogenously across the cellular membrane. The absence of a clear co-localization of transferred ETB-mNG and Rho-PE already gives evidence that lipids and MP separate during the transfer procedure, indicating the disintegration of the NDs. This assumption was further analyzed by immunodetection of MSP. ETB-mNG/NDs (DEPG) and NDs (DEPG +2% Rho-PE) were prepared with Flag-tagged MSP (MSP1E3FN) and incubated with HEK293T cells under standard conditions. After incubation, the cells were permeabilized and stained with anti-Flag antibodies. As a staining control, transfected HEK293T cells expressing GPRC5B-Flag were used. Despite the successful transfer of ETB-mNG and Rho-PE, no MSP1E3FN was detected in the cells by immunostaining (Figure 5A). To further support this result, HEK293T cells after ETB transfer were washed, lysed, and analyzed by western blotting for the presence of ETB and MSP (Figure 5B). The transfer of ETB into the cells was verified, but MSP was only detectable in the transfer mixture and absent in the cell lysate.

### 3.4 MP transfer into HEK293T cells with SapNPs

**FIGURE 6:**
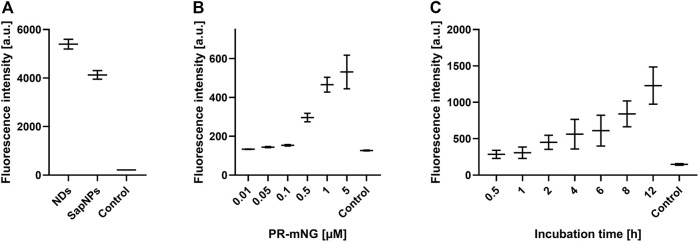
SapNPs as vector for MP transfer into HEK293T cells. PR-mNG/SapNP particles were prepared by CF expression of PR into preformed SapNPs (DOPG). The Strep-purified PR-mNG/SapNPs were incubated with HEK293T cells and the mNG fluorescence in the cellular membrane was quantified. **(A)** Comparison of PR-mNG transfer from NDs (DOPG) and SapNPs (DOPG) at standard conditions. **(B)** Effect of PR-mNG/SapNP concentration on PR-mNG transfer. **(C)** Effect of incubation time on PR-mNG transfer. Mean ± SEM is shown. N = 20 cells. MP-free NDs (DOPG) were used as a negative control.

SapNPs based on the scaffold protein SapA are an alternative option to solubilize MPs in nanomembranes (Frauenfeld et al., 2016). SapA is a sphingolipid activator protein in lysosomes and in contrast to NDs, SapNPs are adaptable to the size of the inserted MP (Chien et al., 2017). This specific characteristic of SapNPs might become interesting for the insertion and solubilization of larger MPs or MP complexes. Although similar in their function to solubilize lipids and MPs, SapA and MSP do not share extensive sequence homologies. We, therefore, analyzed whether SapNPs can be used similarly to NDs as vectors to transfer inserted MPs into living cells. PR-mNG was co-translationally integrated into preformed SapNPs (DOPG) and after Strep-purification incubated with HEK293T cells under standard conditions. After washing and fixation, membrane fluorescence was quantified. As a control, PR-mNG/NDs were incubated with HEK293T cells as well. PR-mNG was transferred from both particles into HEK293T cells (Figure 6A). The PR-mNG transfer from NDs showed a higher efficiency, giving evidence that potentially higher concentrations of MP/SapNPs need to be used in the transfer mixtures in order to obtain comparable MP transfer efficiencies as with NDs. In accordance, a notable transfer of PR-mNG from SapNPs was observed with PR-mNG/SapNP concentrations in the transfer mixture above 0.1 µM (Figure 6B). With NDs, already MP/ND concentrations starting with 0.01 µM resulted in detectable MP transfer (Figure 3B). Similar to the transfer of MPs from NDs, the transfer of PR-mNG from SapNPs continuously increased with prolonged incubation times (Figure 6C).

### 3.5 Membrane topology of transferred MPs

**FIGURE 7:**
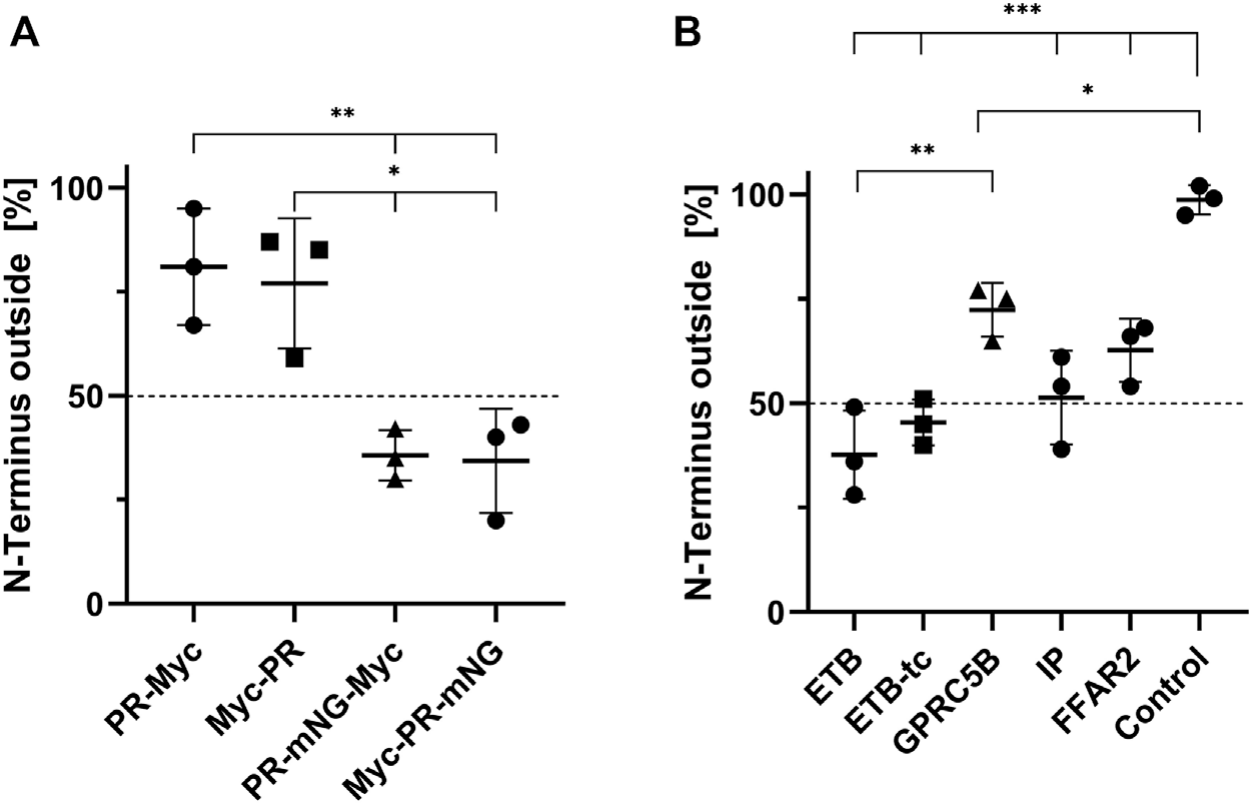
Orientation of transferred MPs in HEK293T cell membranes. NDs containing Myc-tagged MPs were transferred into HEK293T cells at standard conditions. The cells were fixed and either permeabilized with 0.2% Triton X-100 or left with intact cellular membranes. Anti-Myc immunostaining of permeabilized cells resulted in staining of all transferred MPs, while in non-permeabilized cells only MPs with an extracellularly accessibly Myc-tag were stained. The membrane fluorescence was quantified accordingly and the fraction of N-terminally accessible MPs was calculated by dividing the intensity of permeabilized by the intensity of non-permeabilized cells. **(A)** Membrane topology of transferred PR derivatives from NDs (POPG). **(B)** Membrane topology of transferred N-terminally Myc-tagged GPCRs. The transfer mixtures contained Myc-ETB/NDs, Myc-ETB-tc/NDs, Myc-FFAR2/NDs, Myc-GPRC5B/NDs or Myc-IP/NDs. GPRC5B-Myc synthesized in transfected cells was used as a positive control. Mean ± SEM is shown. N = 3. N = 20 cells (*p < 0.05; **p < 0.01; ***p < 0.001; one-way ANOVA with Tukey’s test).

The membrane topology of transferred MPs is of general importance as it strongly affects the MP function. To this point, it was unclear whether MPs insert with a preferred orientation into the cellular membrane or if the insertion is rather random. It seems reasonable that larger soluble domains of an MP might affect its transfer and final membrane topology. Therefore, PR constructs containing a Myc-tag and with or without the large soluble mNG domain either at the N-terminus or at the C-terminus were created. All four constructs were cotranslationally inserted into NDs (POPG), purified, and transferred into HEK293T cells at standard conditions. The position of the Myc-tags was then determined by immunostaining of permeabilized and non-permeabilized cells (Figure 7). The total amount of transferred PR derivatives in the membrane of permeabilized cells was then correlated with the number of transferred PR derivatives in non-permeabilized cells having their Myc-tag only extracellular accessible. The terminal position of the small Myc-tag did not affect the orientation of transferred PR in the HEK293T cell membrane. Approximately 78% of transferred PR-Myc as well as of Myc-PR were oriented with their N-terminus outside, i.e., in the correct and native orientation (Figure 7A). In contrast, fusions of PR with the larger mNG moiety had a strong effect on the insertion orientation and only approx. About 35% of both, PR-mNG-Myc or Myc-mNG-PR, remained with their N-terminal end outside of the cell membrane.

We further analyzed the membrane topology of a number of GPCRs after their transfer from NDs into HEK293T cells. CF prepared NDs containing Myc-ETB, Myc-ETB-tc, Myc-GPRC5B, Myc-FFAR2 or Myc-IP were incubated with HEK293T cells at standard conditions. The membrane topology of the transferred GPCRs in permeabilized and non-permeabilized cells was then determined by immunostaining (Figure 7B). The orientation of Myc-ETB-tc and Myc-IP appears to be rather balanced with approx. 50% having their N-terminal end outside in the correct topology. The truncations of the larger soluble IL3 and C-terminal domains in Myc-ETB-tc seem to have a slight positive effect on its final membrane orientation as only 40% of the full-length Myc-ETB had the correct topology. For Myc-FFAR2 and Myc-GPRC5B a small bias toward the correct membrane insertion with the N-terminus outside was observed. As a control, the membrane topology of GPRC5B-Myc synthesized in transfected HEK293T cells showed a nearly 100% correct N-terminus outside insertion.

### 3.6 Membrane distribution and cluster formation of transferred MPs

**FIGURE 8:**
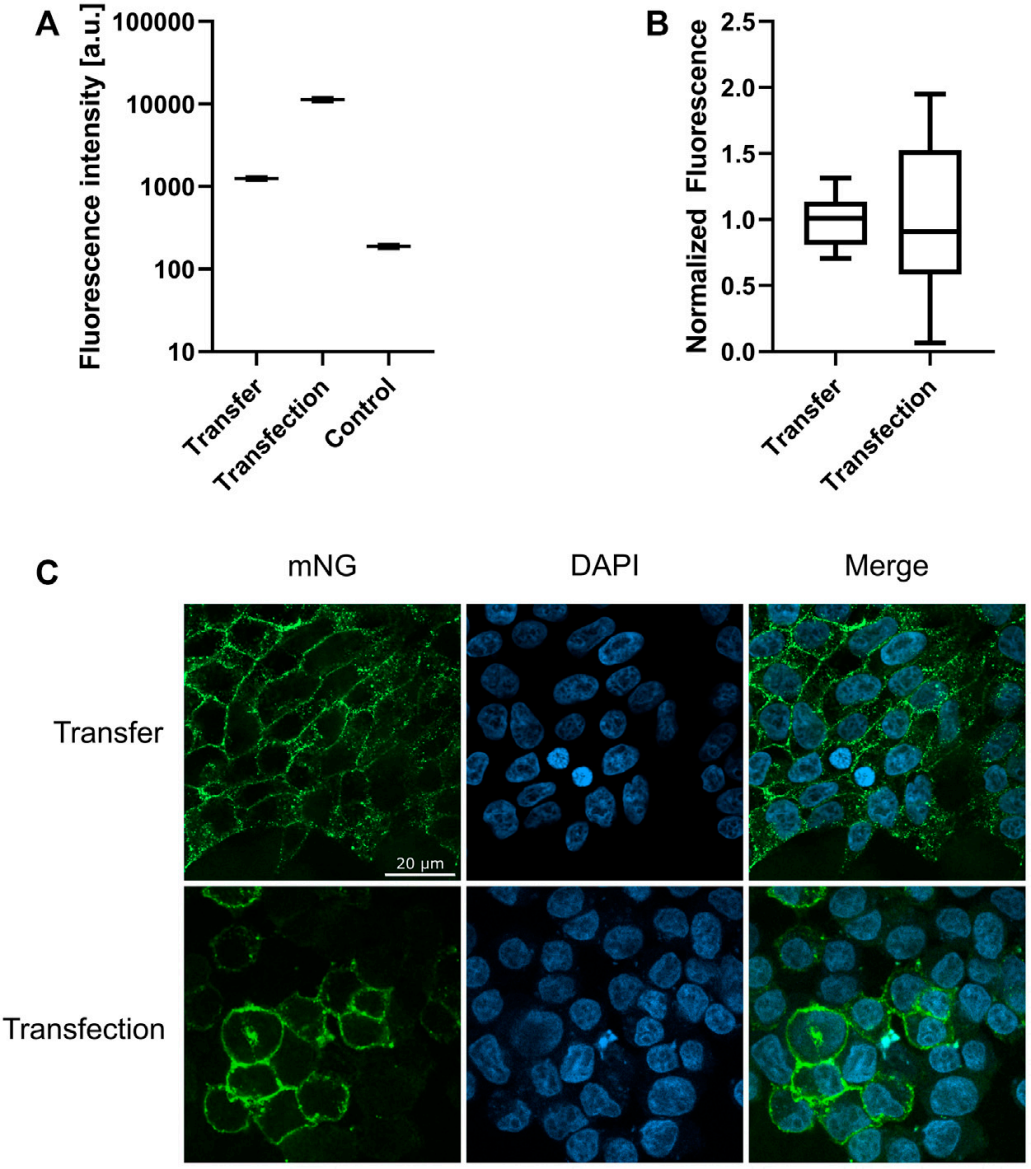
Membrane localization of transferred and transfected ETB-mNG. 0.5 µM ETB-mNG/NDs were incubated with HEK293T cells for 24 h. ETB-mNG was further synthesized in transfected HEK293T cells. Fluorescence microscopy images of fixed cells were taken after 24 h and fluorescence in the cellular membrane was quantified. **(A)** Comparison of membrane fluorescence in transfected and transferred cells. Mean ± SEM is shown. *n* = 20 cells. **(B)** Variation of mean-normalized membrane fluorescence. **(C)** Representative images of HEK293T cells with transferred ETB-mNG or with synthesized ETB-mNG after transfection.

Transfer of ETB-mNG into HEK293T cells as well as the synthesis of ETB-mNG in transfected HEK293T cells results in membrane localization of the GPCR (Figure 8). The total amount of ETB-mNG in transfected cells is approx. 10-times higher if compared with transferred cells (Figure 8A). However, the transferred ETB-mNG is more evenly distributed amongst the transferred cells and all cells in the population have a relatively similar ETB-mNG concentration (Figures 8B,C). In contrast, the ETB-mNG concentration in the transfected HEK293T cell population showed a considerable variation, including cells having very high expression as well as many cells with no detectable ETB-mNG production.

**FIGURE 9:**
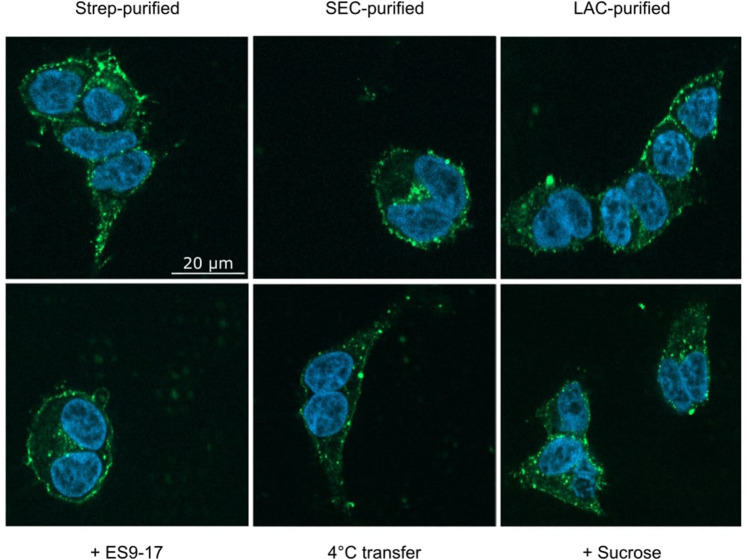
Analysis of cluster formation of transferred ETB-mNG. ETB-mNG/ND particles were purified either (i) via the C-terminal Strep-tag of the receptor, (ii) via the Strep-tag and subsequent SEC, or (iii) via LAC by using immobilized 4Ala-ET-1-biotin. ETB-mNG was then transferred into HEK293T cells at standard conditions and representative fluorescence images of the cells were taken. Lower panel: Effects of addition of the endocytosis inhibitors ES9-17 (30 µM) or sucrose (0.45 M) and of performing the transfer at 4°C on the cluster formation of Strep-purified ETB-mNG.

A striking difference in transfection is the prevalent clustering of transferred MPs in the cell membrane (Figures 2, 4, 8C). While in transfected HEK293T cells the synthesized MP is homogeneously distributed in the cell membranes, with all analyzed MPs a significant fraction of the transferred proteins appeared to be present in clusters. A possible reason for the observed cluster formation could be the transfer of already misfolded and aggregated MPs present in the MP/ND samples. The heterogeneous SEC profiles of all GPCR/ND samples could support this assumption, although even transfer of the homogeneous PR-mNG/ND samples resulted into similar cluster formation. Nevertheless, the effect of sample heterogeneity on the cluster formation was analyzed by transferring ETB-mNG samples of different purity. ETB-mNG/ND particles were either Strep-, SEC-, or LAC-purified. While the Strep-purified sample was the most heterogeneous, containing even larger aggregates, the SEC-purified sample contained mostly fraction 2 with presumably homogeneously inserted ETB-mNG, whereas the LAC-purified sample contained only correctly folded and ligand binding active ETB-mNG. However, the transfer of the differently purified ETB-mNG samples into HEK293T cells did not reveal any difference in membrane localization or cluster formation (Figure 9).

**FIGURE 10:**
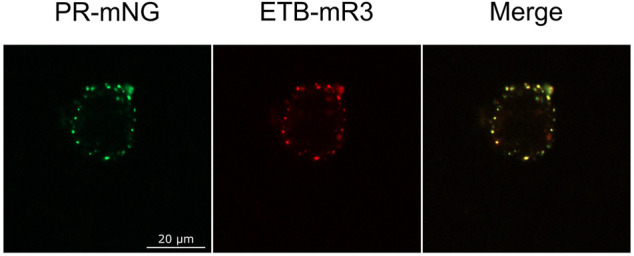
Co-localization of different MP clusters in transferred HEK293T cells. HEK293T cells were co-incubated with PR-mNG/NDs and ETB-mR3/NDs at standard conditions. After washing and fixation, cells were analyzed by fluorescence microscopy. Images of a representative cell are shown.

The observed MP cluster may result from endocytosis processes of the cell. To address this, effects by the addition of the endocytosis inhibitors ES9-17 (Dejonghe et al., 2019) or sucrose (Guo et al., 2015) have been tested. Furthermore, incubation at lower temperatures should also inhibit or retard endocytosis. ETB-mNG was transferred from NDs into HEK cells at standard conditions by pre- and co-incubation with either 30 µM ES9-17 or 0.45 M sucrose, or the transfer mixture was incubated at 4°C. However, none of the modified transfer conditions had a detectable effect on membrane localization or cluster formation of the transferred ETB-mNG (Figure 9). Furthermore, comparable cluster formation in HEK293T cells was independent of the transferred MP type, of the ND lipid composition and it was also visible after MP transfer from SapNPs. Moreover, the cluster formation is fast and already visible after 5 min of incubation (Supplementary Figure S4). The localization of the MP cluster in the cell membrane appears not to be random. HEK293T cells were incubated with PR-mNG/NDs and ETB-mR3/NDs together at standard conditions and analyzed (Figure 10). Cluster formation of both transferred MPs could be monitored individually, and the merged picture showed that most clusters for the two different MPs localize at identical positions in the cell membrane. The results give strong evidence that membrane structures or cellular mechanisms are responsible for the cluster formation, rather than the quality or type of the transferred MP.

### 3.7 Stability and activity of transferred GPCRs

For stability measurements, ETB-mNG transferred HEK293T cells were washed and further incubated in a fresh medium. Fluorescence was measured over a period of 3 days and then gradual decrease of ETB-mNG fluorescence followed a standard protein degradation kinetics (Chen et al., 2016) with a half-life of approx. 15.8 h (Supplementary Figure S5). The transferred cells remained viable and did not show decreased growth rates. The ETB-mNG clusters remained detectable throughout the whole observation period.

**FIGURE 11:**
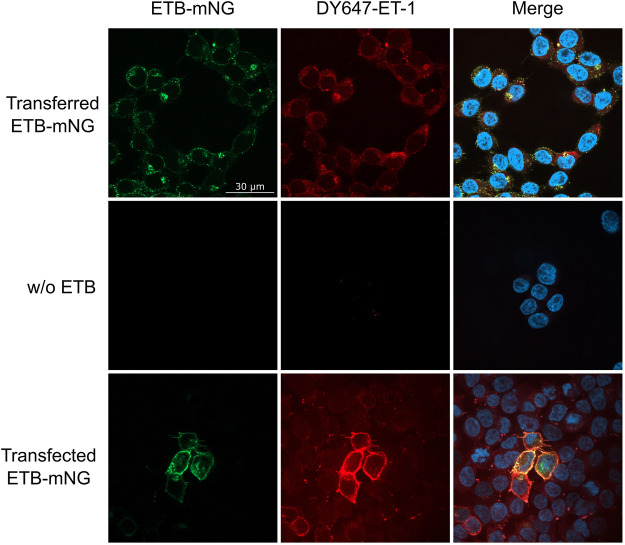
Ligand binding of transferred ETB-mNG in HEK293T cells. LAC-purified ETB-mNG was transferred into HEK293T cells at standard conditions. After washing, cells were incubated with 100 nM DY647-ET-1 for 1 h at 4°C. After additional washing, fluorescence microscopy was performed and representative pictures were taken. Transfected HEK293T cells synthesizing ETB-mNG were used as a positive control.

The functionality of transferred MPs was analyzed by 1) ligand binding, 2) ligand-induced receptor internalization and 3) by specific protein interactions. LAC-purified ETB-mNG was transferred into HEK293T cells at standard conditions. After washing, DY647 labeled ET-1 was added and the cells were further incubated at 4°C to inhibit the internalization of the ligand-receptor complex. Subsequent fluorescence microscopy revealed the co-localization of the transferred receptor with DY647-ET-1, indicating that the functional conformation of ETB-mNG remains unaltered after the transfer (Figure 11). It should be noted that, as shown in Figure 7B, less than 50% of the transferred receptor is inserted with the correct orientation in the membrane. Thus only a fraction of the transferred receptor should be able to bind the ligand. Nevertheless, almost all visible cluster of transferred ETB-mNG show interaction with DY647-ET-1. This gives evidence that the cluster contains mixtures of correct and non-correct inserted receptor and further indicates that cluster formation is not determined by a particular MP structure. As a positive control, DY647-ET-1 was added to HEK293T cells transfected with the corresponding ETB-mNG plasmid, and uniform labeling of membrane-localized ETB-mNG was monitored (Figure 11).

**FIGURE 12:**
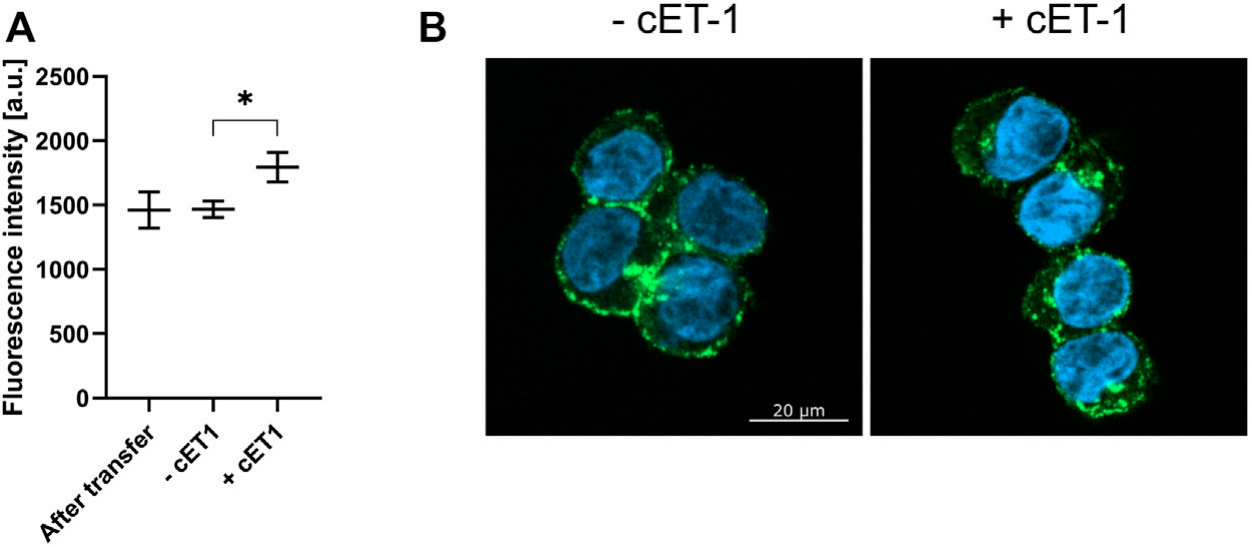
Induced internalization of transferred ETB-mNG by cET-1. LAC-purified ETB-mNG was transferred into HEK293T cells at standard conditions. Cells were washed and subsequently incubated with 500 nM cET-1 for 1 h at 4°C. Afterward, they were further incubated at 37°C for 1 h, fixed and ETB-mNG fluorescence in the cytoplasm was quantified via ImageJ. **(A)** Quantification of ETB-mNG in the cytoplasm of transferred HEK293T cells. Mean ± SEM is shown. *n* = 20 cells (*p* < 0.05; paired *t*-test). **(B)** Representative images of HEK293T cells treated with or without cET-1 after ETB-mNG transfer.

Internalization of transferred LAC-purified ETB-mNG was monitored after incubation with circular ET-1 (cET-1). A primary function of the ETB receptor in human tissue is to efficiently remove endothelin agonists by undergoing rapid internalization upon the formation of an ETB/agonist complex (Wu-Wong et al., 1995; Zrein et al., 2020). ETB-mNG transferred cells were incubated with 500 nM of the native agonist cET-1 for 1 hour and then further incubated at 37°C for 1 hour to allow for internalization. The amount of ETB-mNG in the cytosol of cET-1 stimulated and non-stimulated cells were then quantified after fixation (Figure 12). A significant increase of ETB-mNG fluorescence in the cytosol fraction after cET-1 stimulation gives further evidence of its functional integration into the cellular membrane environment after the transfer process.

**FIGURE 13:**
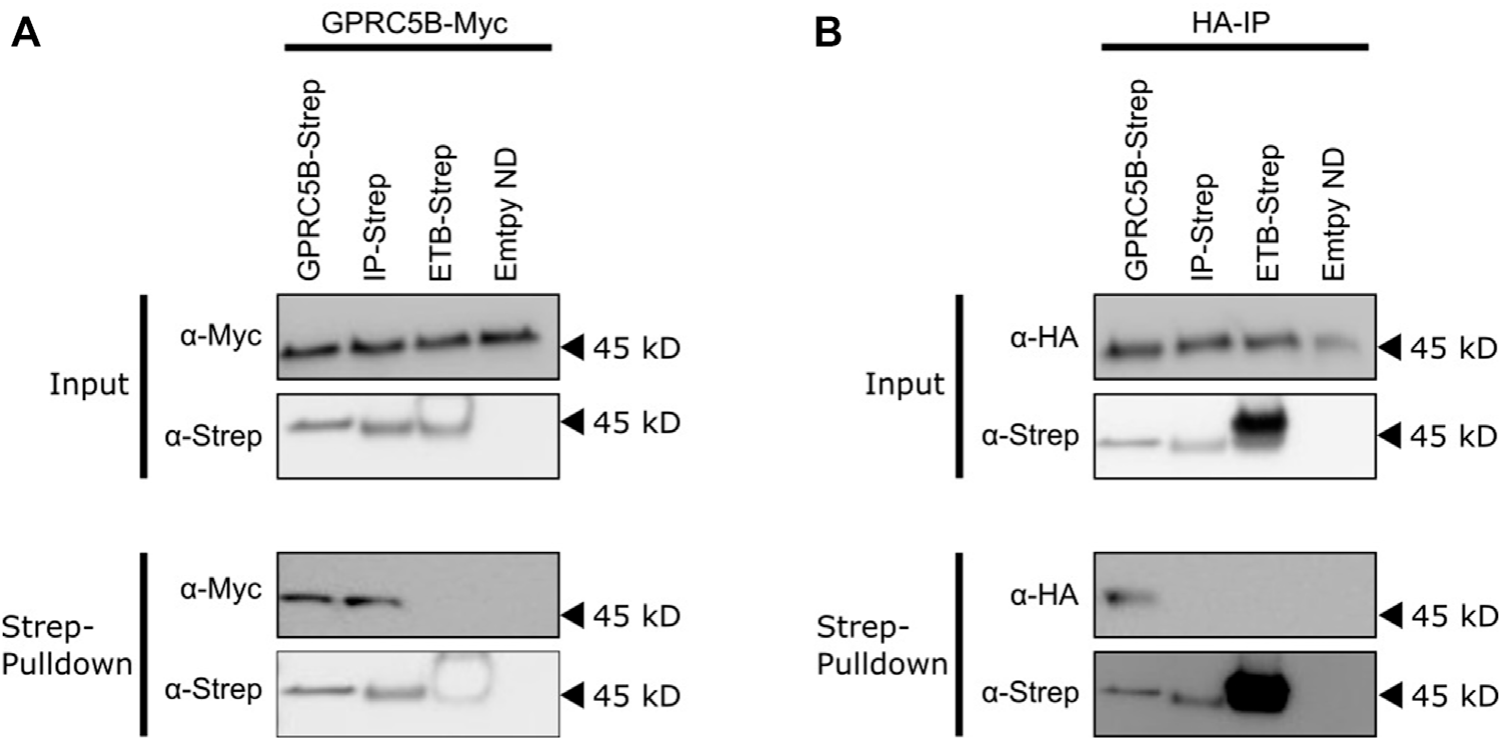
Interactions of transferred and transfected GPCRs. HEK293T cells were transfected with constructs expressing either GPRC5B-Myc **(A)** or HA-IP **(B)**. After 24 h, the transfected cells were incubated with 0.5 µM of either GPRC5B/NDs, IP/NDs, ETB/NDs or empty NDs for 16 h to allow efficient MP transfer. The cells were then washed, lysed and pulldowns based on the Strep-tags of the transferred GPCRs were performed. The MPs were subsequently analyzed for co-purification by SDS-PAGE and immunoblotting. **(A)** Analysis of transfected cells synthesizing GPRC5B-Myc. Upper panel input: Immunoblotting of cell lysates showing successful expression of GPRC5B-Myc (α-Myc) and the successful transfer of either GPRC5B, IP or ETB (α-Strep). Lower panel Strep-pulldown: Immunoblotting of the eluate from the Strep-column detecting transferred GPCRs (α-Strep) and co-purified transfected GPRC5B-Myc (α-Myc). No pulldown of GPRC5B-Myc was observed with transferred ETB or empty NDs. **(B)** Analysis of transfected cells synthesizing HA-IP. Upper panel input: Immunoblotting of cell lysates with anti-HA antibody shows a successful expression of HA-IP (α-HA) and the successful transfer of either GPRC5B, IP, or ETB into the cells (α-Strep). Lower panel Strep-pulldown: Immunoblotting of the eluate from the Strep-column detecting transferred GPCRs (α-Strep) and co-purified transfected HA-IP (α-HA).

The oligomerization of GPCRs and their interaction with other binding partners requires their functional conformation. The homo-oligomerization of GPRC5B and IP receptor as well as the hetero-oligomerization of GPRC5B with the IP receptor has been described before (Giguère et al., 2004; Carvalho et al., 2020). Strep-tagged GPRC5B or IP receptors were transferred into HEK293T cells previously transfected with constructs encoding for HA-IP or GPRC5B-Myc. After transfer, pulldown experiments based on the Strep-tag of the transferred receptors were performed. Both transferred GPRC5B and transferred IP were interacted with the synthesized GPRC5B-Myc (Figure 13). Furthermore, an interaction of transferred GPRC5B with the HA-IP receptor was detectable. However, no pulldown of transferred IP receptor with HA-IP receptor synthesized after transfection was observed.

## 4 Discussion

The nanotransfer of MPs into live mammalian cells is an emerging tool that complements established techniques for the transfer of soluble proteins (Patriarchi et al., 2018; Chau et al., 2020; He et al., 2021). In combination with CF expression, MP/ND samples ready for transfer can be generated within 24 h. We have analyzed the nanotransfer of four human GPCRs and of PR also shows a seven-transmembrane domain structure. The CF synthesis of the ETB receptor and its truncated derivative ETB-tc into NDs was previously optimized. This work is the first description of the cotranslational insertion of the FFAR2, IP, and GPRC5B receptors into NDs. After optimization, all GPCR/ND particles were synthesized in µM concentrations, but still showed heterogeneous SEC profiles including fractions of functionally folded protein as well as potentially unfolded and aggregated fractions. The best quality was obtained with PR/ND samples showing a homogenous SEC profile.

MP transfer from nanoparticles into the cellular plasma membrane is a rapid process and is already detectable after a few minutes of incubation. The transfer is even much faster than previously reported and different detection techniques might account for this discrepancy (Patriarchi et al., 2018). Transcription, translation, and trafficking processes necessary for MP production in transfected cells are skipped and the recombinant MP appears almost instantly in the cellular plasma membrane. Furthermore, the MP transfer efficiency increases linearly with the incubation times of MP/NPs and cells for a period of several hours. Transfer efficiency further depends on the MP particle concentration in the transfer mixture. This offers an interesting tool to modulate the final concentration of the transferred MP in the cell. Effects of increasing MP concentrations could be determined and potential interaction partners in the cells could be identified and quantified by pulldown assays. The nanoparticle lipid composition appears to be of less importance for the MP transfer efficiency. This is an important finding as it allows us to integrate the synthesized MPs always into their most preferred lipid environment. The nature of the nanoparticle scaffold protein is also of less importance for the MP transfer efficiency. ND as well as SapA particles are suitable vectors for MP transfer. However, MP transfer from SapNPs appears to be a little less efficient than from NDs. This could be explained by the smaller membrane area in SapNPs potentially resulting in a weaker alignment of the SapNPs with the cell membrane (Lai et al., 2015). We could further demonstrate that NDs dissociate during transfer and that the transferred lipids and MPs separate from each other in the cell membrane. The scaffold protein MSP stays outside of the cells and we did not find any evidence of MSP uptake. This disagrees with previous reports showing uptake of fluorescent labeled MSP from HeLa cells after their incubation with NDs (Carney et al., 2015; Petrache et al., 2016). The labeled MSP was localized as punctuate spots inside the cells. However, labeled lipids from NDs are dispersed throughout the cell membrane, indicating a dynamic exchange of lipids between NDs and cells similar to our observations. It can therefore be concluded that NDs disintegrate upon transfer and release the lipids into the cell membranes, whereas a partial uptake of the MSP might be cell type-specific.

While the MP transfer appears to be possible with a variety of cell lines, we provide the first evidence that differences may exist with regard to MP localization and stability after transfer. In our hands, the plasma membrane localization of transferred MPs was most prominent in HEK293T cells and somehow less pronounced in H1299 cells. With CHO cells, most of the transferred MPs are localized in the cytoplasm. It has been shown that the composition of supported lipid bilayers affects the transfer of MPs from NDs (Dang et al., 2019). Different membrane compositions of the analyzed cell lines might therefore contribute to the observed preference in MP localization.

The transfer mechanism appears to be more general as similar efficiencies were obtained with all five analyzed MPs. According to the fusion pore model for the transfer of cargo from NDs to bicelles, the NDs are suggested to align in a planar orientation on top of the target bilayer (Lai et al., 2015). The MPs may then spontaneously transfer between nanoparticle and cell membranes (Dang et al., 2019). Two contact sites of the MP/ND particles with the cell membrane are possible. Our results indicate that the membrane insertion of MPs with comparable topology is rather stochastic without a notable preferred orientation. However, steric hindrance by the presence of larger soluble domains could have a strong effect on the resulting MP membrane topology by causing an orientation bias of the aligned MP/ND particles. While a small soluble Myc-tag terminally attached to PR did not affect its orientation after membrane insertion, fusion of the larger mNG moiety to the PR C-terminus reduced the amount of “correctly” inserted MP (= N-terminus outside) from approx. 75% to less than 30%. It is important to note that the insertion of MPs into cellular plasma membranes with the wrong orientation is a distinct feature of the nanotransfer technique and might be interesting for particular experimental approaches. MPs produced from transfected cells are targeted to the plasma membrane exclusively in their correct orientation.

After prolonged incubation of 4 hours, the amount of transferred MP in HEK293T cells was still significantly lower if compared with transfected cells. However, the recombinant production of MPs in DNA transfected cells is hard to control and high variation exists with regard to the level of synthesized protein in individual cells (Kim and Eberwine, 2010). In accordance, a fraction of the HEK293T cells transfected with a DNA template of ETB-mNG showed high production of the recombinant MP, whereas a significant number of cells had no detectable ETB-mNG production at all. In contrast, the distribution of transferred ETB-mNG in HEK293T cells was much more homogenous within the cell population and cells without any transferred MP were hardly detectable. More homogenous MP production in a cell population may be obtained by transfection with RNA-based templates, while the requirement for specific RNA regulatory structures and difficult handling techniques still can cause problems (Kim and Eberwine, 2010). The nanotransfer approach could thus become interesting for approaches, where homogenous distribution and comparable MP concentrations within a cell population are more relevant than a total high protein concentration.

A striking difference in MP production in transfected cells is the accumulation of transferred MPs in clusters, while transferred lipids distribute homogeneously among the cellular plasma membrane. The cluster formation was generally visible with all analyzed MPs to similar extents. It was not due to the transfer of already aggregated MPs in the case of the GPCR samples, as control experiments with LAC-purified ETB-mNG gave similar results. In addition, also transfer of homogeneous PR samples resulted in cluster formation. Most interesting is the co-localization of cluster formed with different transferred MPs. Since clusters are visible already after a few minutes of transfer, they might show “hot spots” where the transfer of the MP from the ND takes place, indicating that the transfer might be dependent on certain microdomains in the cellular membrane. Alternatively, transferred MPs could diffuse to defined membrane areas where they become immobilized by certain mechanisms. Ligand binding indicated that the cluster appears to contain functionally folded MPs rather than unfolded aggregates. The cluster did not resolve during incubation and we could not find evidence for an involvement in endocytosis processes. It will be interesting to compare the cluster formation of transferred MPs in other cell lines in order to further reveal mechanistic details.

The transferred GPCRs show standard degradation kinetics and were detectable in the cells for approx. 48 h. The functional transfer of ETB was shown by binding to its ligand ET-1 and by the stimulation of ETB internalization after binding to the ligand cET-1. This is in accordance with previous reports showing signaling activation by transferred human ß2-adrenergic receptors (Patriarchi et al., 2018). We further demonstrated the interaction of transferred GPCRs with their cognate binding partners synthesized after transfection of the same cells. The GPRC5B receptor synthesized in transfected cells interacted with both, CF synthesized and transferred GPRC5B or IP receptor. This is in agreement with the previously observed homodimerization of GPRC5B and heterodimerization of GPRC5B with the IP receptor (Carvalho et al., 2020). Vice versa, the IP receptor synthesized in transfected cells interacted with CF synthesized and transferred GPRC5B, while interaction with the transferred IP receptor was not observed. Interestingly, although the IP homo-oligomerization has been described before, it is speculated that this interaction is based on intermolecular disulfide bonds formed already in intracellular compartments (Giguère et al., 2004). The failure to observe an interaction of transferred with transfected IP may therefore result from the inability of the transferred MP to form these intermolecular covalent bonds in the cellular membrane. In addition to the mentioned examples of transferred functional GPCRs, a recent report demonstrated the alteration of the cellular transcriptome and induction of a tumor-like phenotype in originally non-malignant breast cells transferred with the receptor tyrosine kinase Her2 (He et al., 2021).

In conclusion, CF expression, nanoparticle technology and nanotransfer combine in excellent synergy to generally allow the fast insertion of recombinant MPs into membranes of live cells. The final MP amount in the transferred cells can be fine-tuned by incubation time and initial MP nanoparticle concentration, allowing for a more precise adjustment of recombinant MP in a cell population. The process is detergent-free and does not require potentially toxic chemicals, hazardous vectors or physical manipulation of the cells. The technique has a lower impact on cell physiology and might therefore in particular be suitable for sensitive, fragile or otherwise difficult to transfect cells such as primary cells, stem cells, cancer cell lines or stationary phase cells (Patriarchi et al., 2018; He et al., 2021). The speediness can induce immediate cellular reactions allowing the simultaneous *in vitro* and *in vivo* analysis of aliquots of identical samples. In addition to basic research applications, the nanodelivery approach might have potential for therapeutic studies as well. NDs were shown to be non-toxic and non-immunogenic in mice and displayed high stability in biological fluids (Fischer et al., 2014). Cell-type specific targeting might be addressed in the future by using chimeras of MSP and cell-specific antibody fragments (Crosby et al., 2015). It needs to be considered that, in contrast to MPs synthesized after transfection, the transferred MPs insert in more or less random orientation in the cell membranes, while engineering strategies may generate a bias in the orientation. Furthermore, the transferred MPs will be devoid of posttranslational modifications and are predominantly organized in membrane-located clusters. A variety of functions including ligand binding, protein interactions, and signaling are documented from transferred MPs, but the effects of this cluster formation on MP functionality can currently not be excluded.

## Data Availability

The original contributions presented in the study are included in the article/supplementary material; further inquiries can be directed to the corresponding author.
