# Supplementary material for: Transfer mechanism of cell-free synthesized membrane proteins into mammalian cells
Source: Front Bioeng Biotechnol. 2022 Jul 22;10:906295. doi: 10.3389/fbioe.2022.906295 (PMC9355040; doi:10.3389/fbioe.2022.906295)
Supplement: Supplementary file 1 [file DataSheet1.PDF]

Supplementary Materials

Table S1. List of constructs

Construct	Size [kDa]	N-tag	C-tag	Fluorophore
ETB	43.8	H	StrepII	-
ETB-mNG	75.5	H	StrepII	mNeonGreen
ETB-tc-mNG[a]	T, 70.7	H	StrepII	mNeonGreen
Myc-ETB	49.8	Myc + H	StrepII	-
Myc-ETB-tc[a]	45	Myc + H	StrepII	-
ETB-mNG[b]	87.1	-	-	mNeonGreen
PR-mNG	53.5	H	StrepII	mNeonGreen
PR-Myc	28	H	Myc + StrepII	-
Myc-PR	28	Myc + H	StrepII	-
PR-mNG-Myc	54.7	H	Myc + StrepII	mNeonGreen
Myc-PR-mNG	54.7	Myc + H	StrepII	mNeonGreen
GPCR5B	46.7	H	StrepII	-
Myc-GPRC5B	47.8	Myc + H	StrepII	-
GPRC5B-mNG	73.6	H	StrepII	mNeonGreen
GPRC5B-MycFlag[c]	44.8	-	Myc + Flag	-
IP	42.6	H	StrepII	–
Myc-IP	43.8	Myc + H	StrepII	-
IP-mNG	69.5	H	StrepII	mNeonGreen
HA-IP[c]	44.3	3x HA	-	-
FFAR2-mNG	65.9	H	StrepII	mNeonGreen
Myc-FFAR2	40	Myc-H	StrepII	-

a, ETB-tc C-terminally truncated after L401
b, wild type ETB with signal sequence, for eukaryotic expression, T. Meckel
c, for eukaryotic expression, N. Wettschureck

Preparation of ET-1 ligands

All chemicals for synthesis, including standard Fmoc amino acids, coupling reagents, resins, solvents, and all other reagents, were obtained from Orpegen, Iris, Novabiochem, RAPP Polymere, Acros, or Aldrich. Analytical high-performance liquid chromatography (HPLC) experiments were performed on a Hitachi Primaide HPLC system (column: Nucleodur C_{18}, 5 µm, 100 Å, 4.6 x 250 mm, Macherey–Nagel, Düren, Germany) with a linear gradient of acetonitrile (20–70% in 50 min, detection at 220 nm) in water both containing 0.1% trifluoroacetic acid (TFA) at a flow rate of 1 mL/min. The final peptides were purified by a preparative Knauer Azura HPLC system (pumps: P2.1L; detector: UVD2.1L; fraction collector: Foxy R1/R2) using a C_{18} column (Nucleodur, 5 µm, 100 Å, 32 x 250 mm, Macherey–Nagel) and a linear gradient of acetonitrile in water both containing 0.1% TFA at a flow rate of 20 mL/min. All peptides were finally obtained as TFA salts after lyophilization. The molecular mass of the synthesized compounds was determined using a QTrap 2000 electrospray ionization (ESI) spectrometer (Applied Biosystems).

The automated solid phase peptide synthesis was performed on a Syro 2000 peptide synthesizer (MultiSynTech, Witten, Germany) using approximately 90 mg of preloaded Fmoc-Trp(Boc)-Wang resin (loading 0.29 mmol/g) for each 2 mL reaction vessel and a standard Fmoc protocol with double couplings (≈4-fold excess of Fmoc-amino acid, HOBt, and HBTU, respectively, and 8 eq of DIPEA, 2 × 2 h coupling time) using DMF as solvent. The resins with the protected peptides 4-Ala-ET-1 ([Ala^{1,3,11,15}]ET-1) and 4-Ala-Arg9-ET-1 (additional substitution of Lys9 by Arg) were used for the following reactions.

For the preparation of the biotinylated 4-Ala-ET-1, two lysine residues were initially coupled via their side chains as a flexible linker segment. Therefore, after removal of the N-terminal Fmoc protection, Boc-Lys(Fmoc)-OH was manually attached twice to the protected 4-Ala-ET-1 resin by single couplings with 3-fold excess of amino acid, HOBt and HBTU in presence of 6 eq DIPEA in DMF (2 h coupling time). After Fmoc removal, a 10-fold excess of biotin, HOBt and HBTU in presence of 20 eq DIPEA in a 1:1 mixture of DMF/DMSO was used for coupling (17 h coupling time). The peptide was cleaved from resin and deprotected by a mixture of TFA/triisopropylsilane/water (95:2.5:2.5, *v*/*v*/*v*) over 2.5 h at room temperature and precipitated in cold diethyl ether. The crude peptide (partially oxidized methionine) was reduced with 0.725 M N-methylmercaptoacetamide in water for 72 h (Houghten and Li, 1979) and subsequently purified by preparative reversed-phase HPLC (purity 89.1% based on detection at 220 nm). After final lyophilization, the peptide was obtained as TFA salt (3.1 mg of white solid; HPLC: 22.4 min; ESI-MS: calc. 2848.4, found *m*/*z*: 1425.9 $(M+2H)^{2+}$).

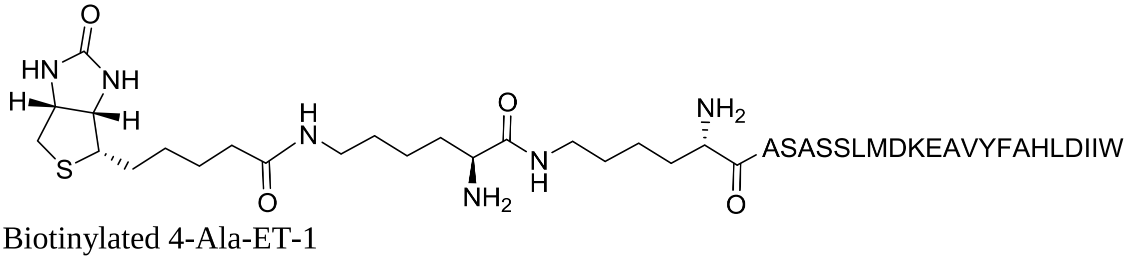

Biotinylated 4-Ala-ET-1

For preparation of derivative DY647-4-Ala-Arg9-ET-1, the 4-Ala-Arg9-ET-1 was cleaved from resin and deprotected by a mixture of TFA/triisopropylsilane/water (95:2.5:2.5, *v*/*v*/*v*) over 2.5 h at room temperature and precipitated in cold diethyl ether. The crude peptide was reduced with 0.725 M N-methylmercaptoacetamide in water for 72 h. The 4-Ala-Arg9-ET-1 was purified by preparative HPLC and lyophilized (HPLC: 26.8 min). 3.0 mg (1.3 µmol, 1.0 eq) of this intermediate was dissolved in 600 µL DMF and treated with 1.0 mg

(1.3 µmol, 1.0 eq) DY-647-NHS-ester (Dyomics GmbH, Jena, Germany) in presence of 6 eq DIPEA. The mixture was shaken for 23 h at room temperature. Afterwards, the peptide was purified by preparative HPLC and obtained as lyophilized TFA salt (0.5 mg of blue solid; HPLC: 26.8 min; ESI-MS: calc. 3084.4, found *m*/*z*: 1544.5 $(M+2H)^{2+}$).

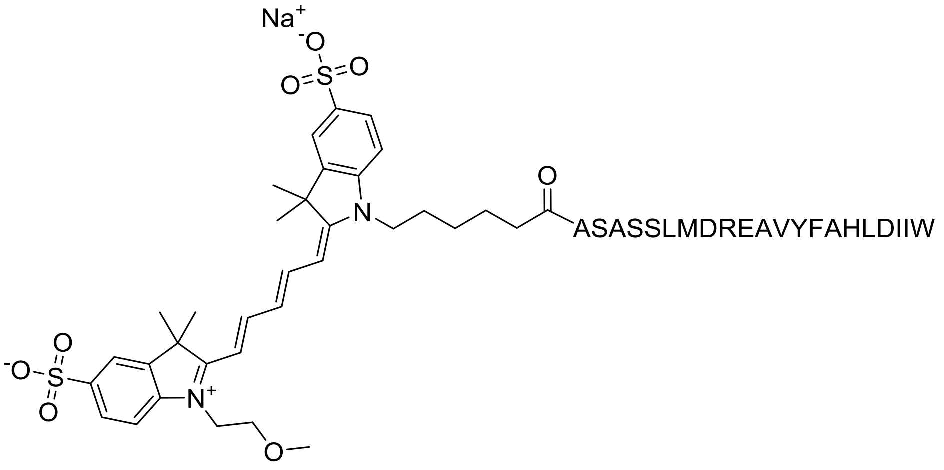

DY647-4-Ala-Arg9-ET-1

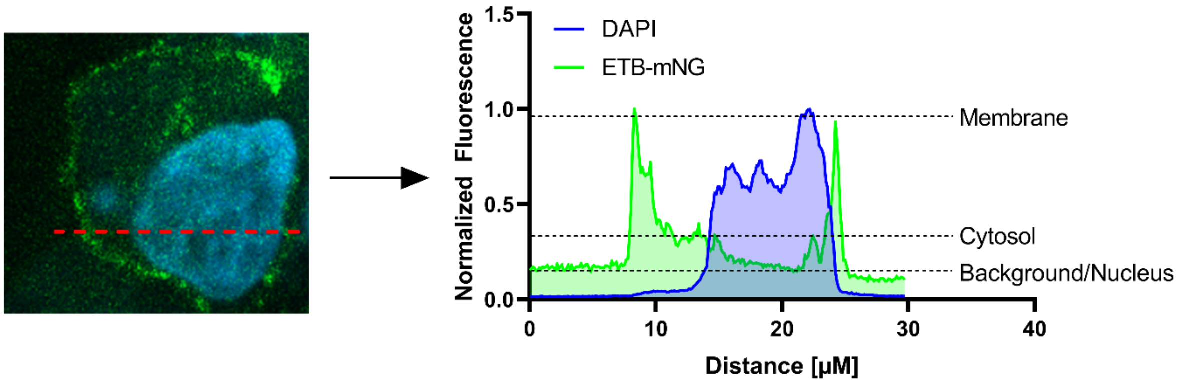

Fig. S1. Localization of transferred ETB-mNG in the HEK293T plasma membrane. Representative image of a HEK293T cell after transfer of ETB-mNG at standard conditions. The fluorescence profile in the right diagram is taken according to the indicated red line of the cell.

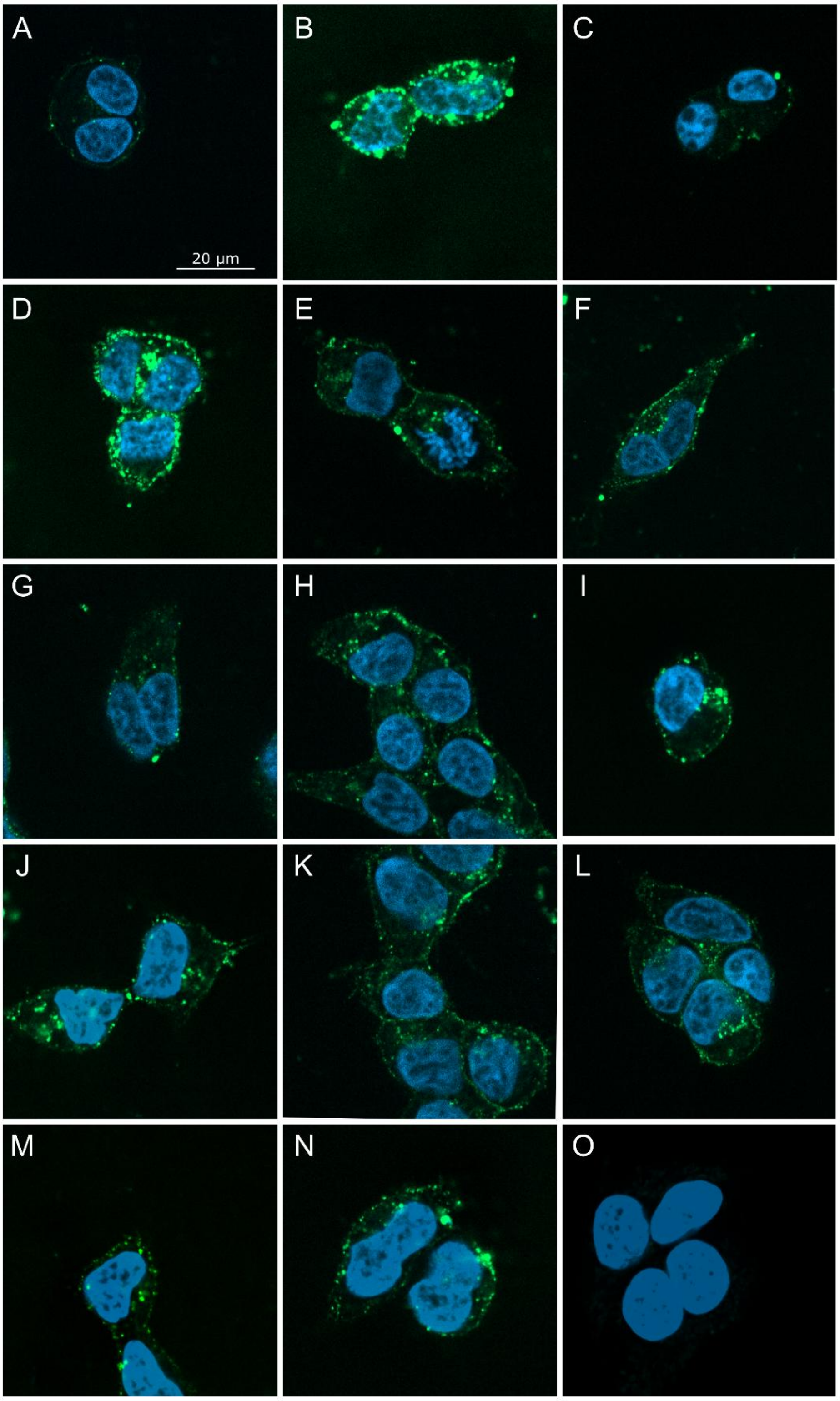

Fig. S2. Representative fluorescence microscopy images of analyzed nanotransfer conditions corresponding to Fig. 3. Strep-purified MP-mNG/ND complexes where transferred into HEK293T cells. Fig. 3A: **A**: 0.5 µM ETB-mNG/ND, 1 h. **B**: 0.5 µM ETB-mNG, 24 h. Fig. 3B: **C**: 0.01 µM ETB-mNG, 4h. **D**: 10 µM ETB-mNG, 4h. Fig. 3C: **E**: 0.5 µM PR-mNG in DEPG NDs, 4h. **F**: 0.5 µM PR-mNG in POPG NDs, 4h. **G**: 0.5 µM PR-mNG in DMPC NDs, 4h. **H**: 0.5 µM PR-mNG in DOPG NDs, 4h. Fig. 3D: **I**: 0.5 µM ET-tc-mNG, 4h. **J**: 0.5 µM ETB-mNG, 4h. **K**: 0.5 µM FFAR2-mNG, 4h. **L**: 0.5 µM GPRC5B-mNG, 4h. **M**: 0.5 µM IP-mNG, 4h. **N**: 0.5 µM PR-mNG, 4h. **O**: 0.5 µM POPG NDs without MP.

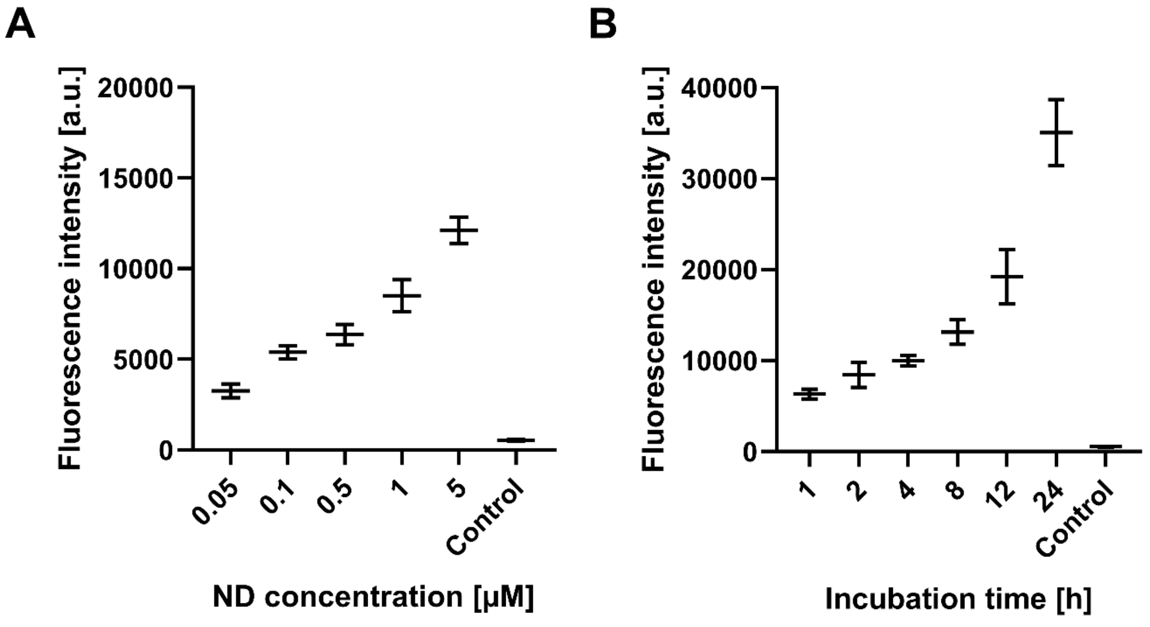

Fig. S3. Effect of particle concentration and incubation time on lipid transfer into HEK293T cells. NDs (DEPG + 2% Rho-PE) were incubated with HEK293T cells at standard conditions with the indicated modifications. Afterwards, cells were washed, fixed and membrane fluorescence was quantified. **A**: Effect of ND particle concentration on lipid transfer. **B**: Effect of incubation time on lipid transfer. Mean ± SEM is shown. n = 20 cells. Controls are fluorescence of HEK293T cells after incubation with NDs without Rho-PE lipids.

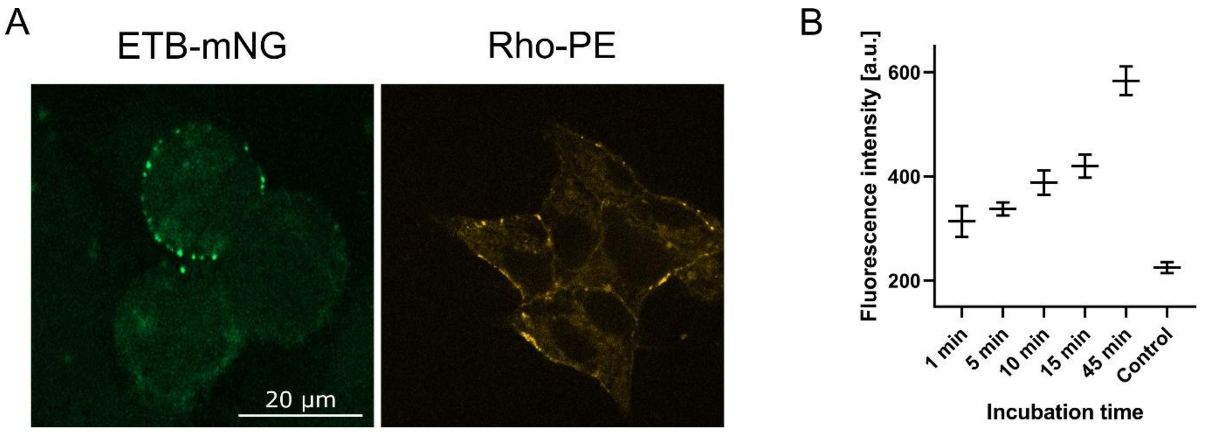

Fig. S4. Fast transfer of MP or lipids from NDs into HEK293T cells. A: Representative images of cells 5 min after transfer of ETB-mNG or Rho-PE lipids at standard conditions. **B**: Fluorescence detection in the cell membrane within the first 45 min after transfer of ETB-mNG at standard conditions. Mean ± SEM is shown. n = 20 cells. MP-free NDs were used as negative control.

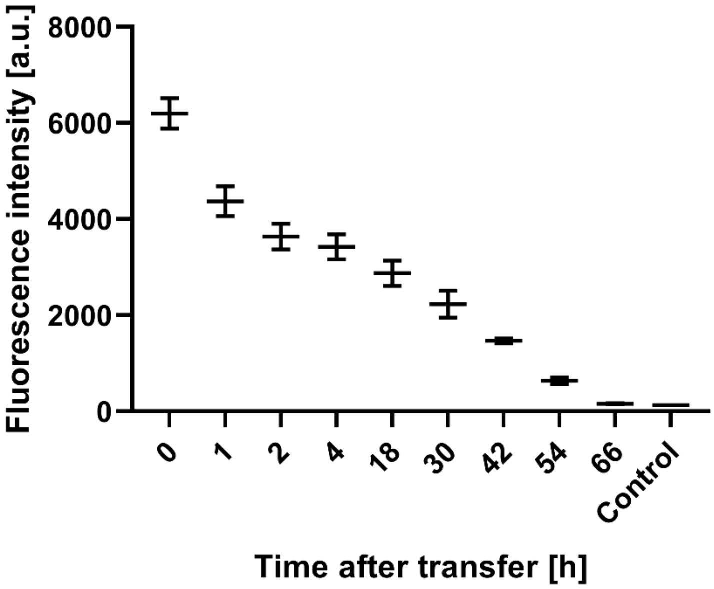

Fig. S5. Degradation kinetics of transferred ETB-mNG. ETB-mNG was transferred into HEK293T cells at standard conditions and further incubated in fresh medium. Membrane fluorescence was then determined over a period of 66 hours. Mean ± SEM is shown. n = 20 cells.

References

Houghten, R. A., and Li, C. H. (1779). Reduction of sulfoxides in peptides and proteins. Anal. Biochem. 98 (1), 36-46.

Abbreviations

Boc, tert-butoxycarbonyl; DIPEA, diisopropylethylamine; DMF, N,N-dimethylformamide; DMSO, dimethyl sulfoxid; ET, Endothelin; Fmoc, N-(9-fluorenyl)methoxycarbonyl; HBTU, (2-(1H-benzotriazol-1-yl)-1, 1, 3, 3-tetramethyluronium hexafluorophosphate; HOBt, N-hydroxybenzotriazole; HPLC, high performance liquid chromatography; MS, mass spectrometry; TFA, trifluoroacetic acid
